# Supplementary material for: Revisiting the Phylogenetic Relationship and Evolution of Gargarini with Mitochondrial Genome (Hemiptera: Membracidae: Centrotinae)
Source: Int J Mol Sci. 2022 Dec 30;24(1):694. doi: 10.3390/ijms24010694 (PMC9821036; doi:10.3390/ijms24010694)
Supplement: Supplementary file 1 [file ijms-24-00694-s001.zip › Figures S1-S11.pdf]

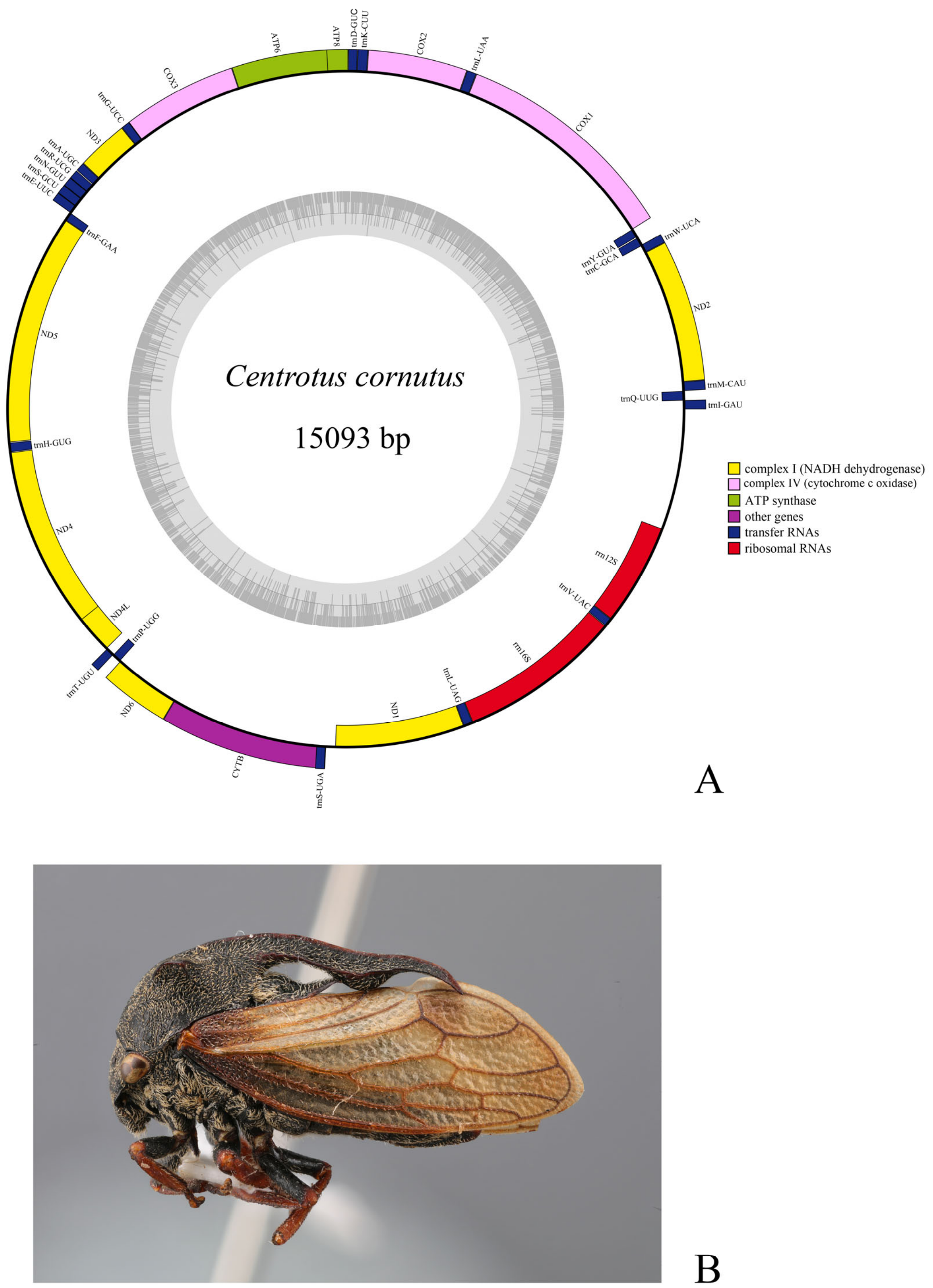

Figure S1. A: Circular maps of the mitogenomes of the species *Centrotus cornutus*; B: the lateral view of *Centrotus cornutus*.

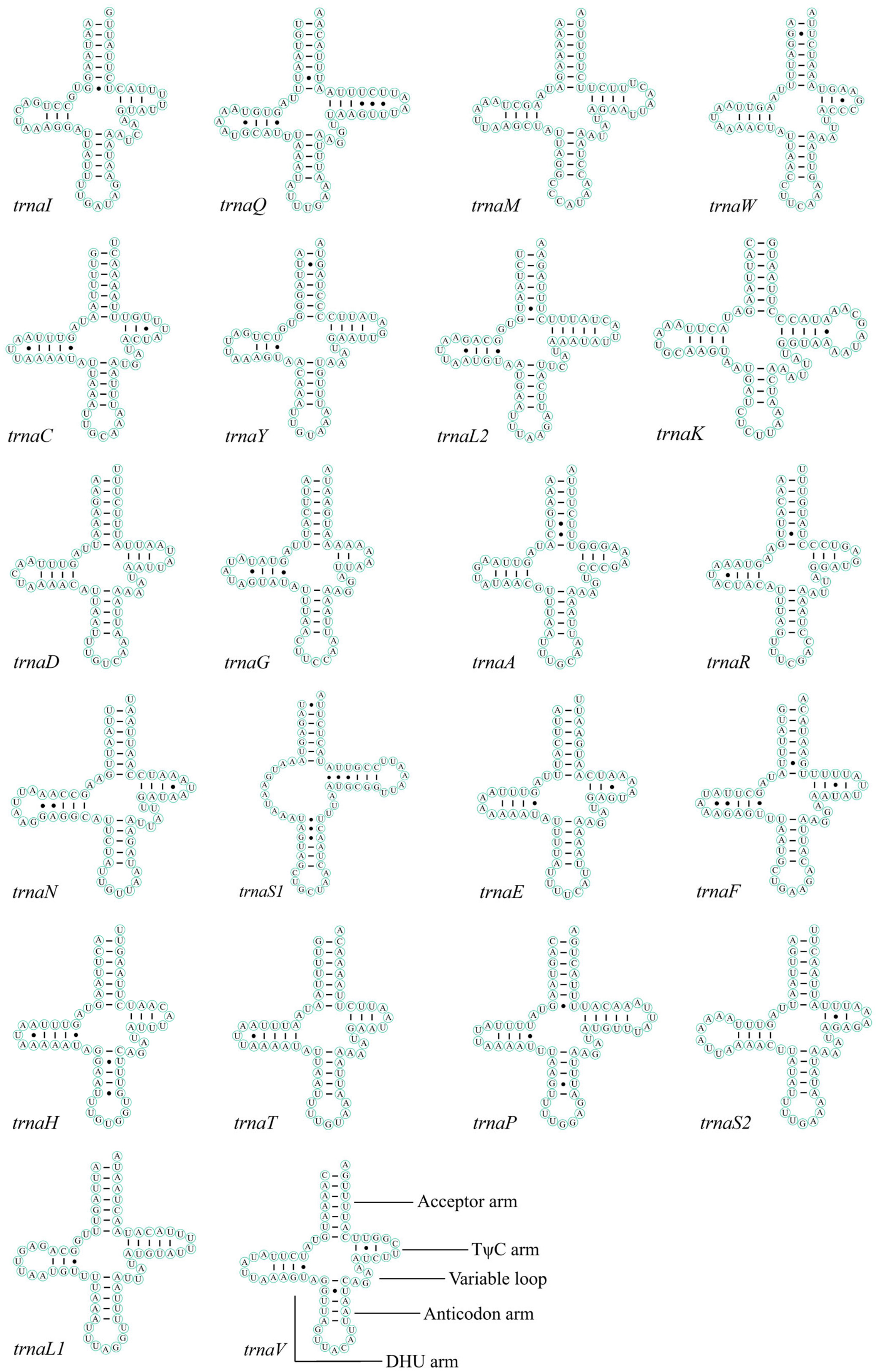

Figure S2. Predicted secondary cloverleaf structure for the tRNAs of *Centrotoscelus davidi*

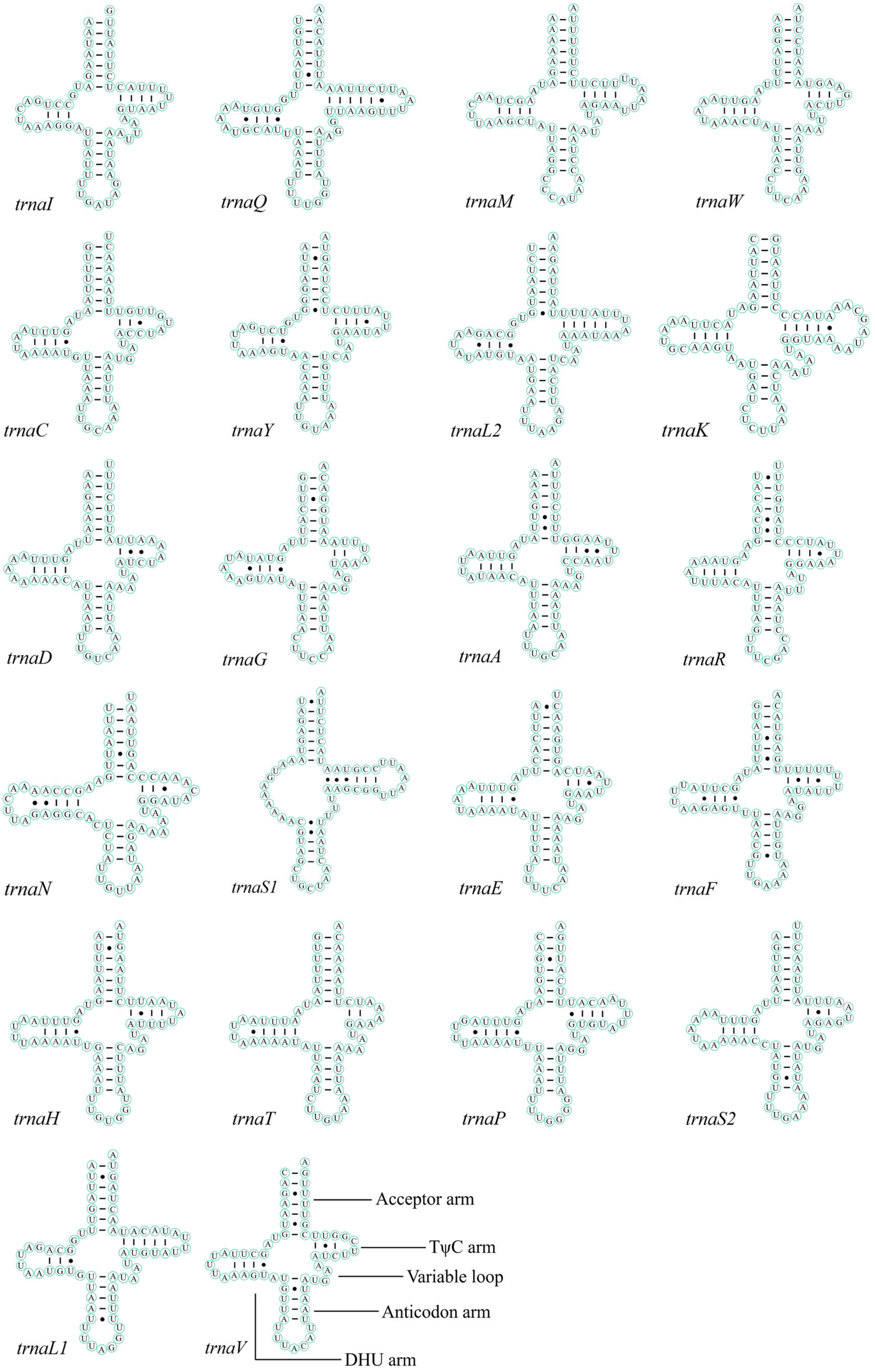

Figure S3. Predicted secondary cloverleaf structure for the tRNAs of *Kotogargara minuta*

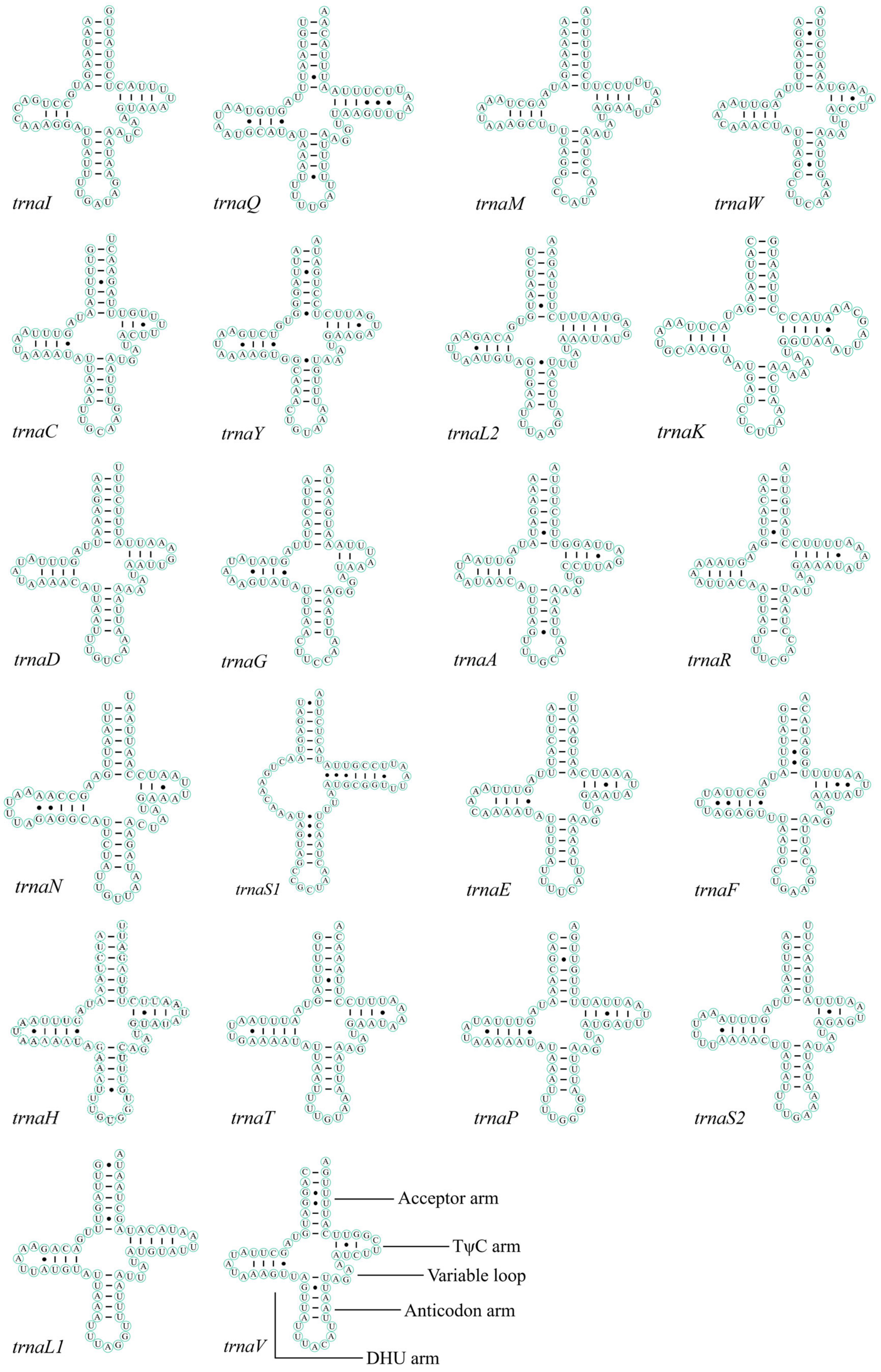

Figure S4. Predicted secondary cloverleaf structure for the tRNAs of *Tricentrus fulgidus*

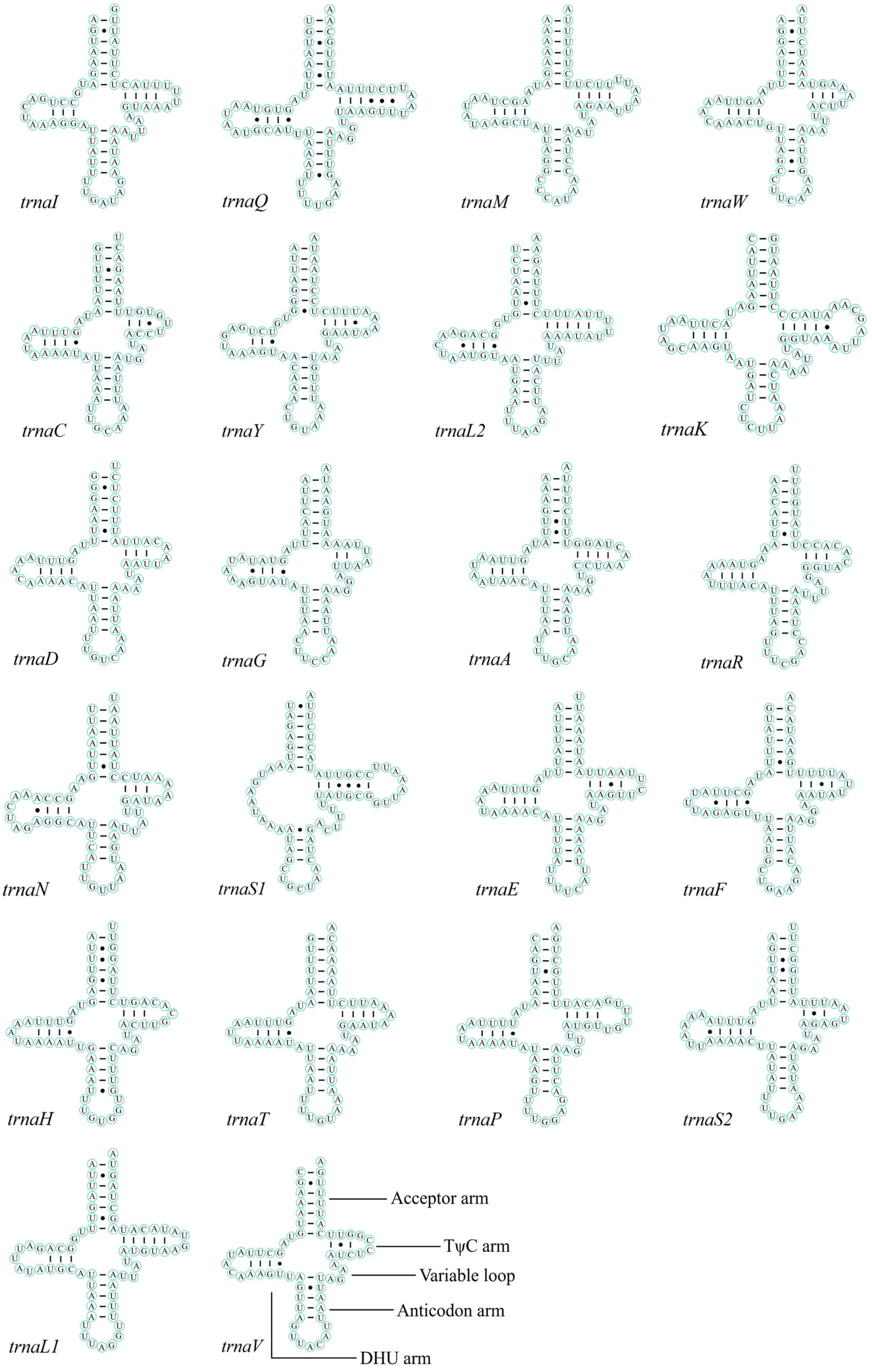

Figure S5. Predicted secondary cloverleaf structure for the tRNAs of *Tricentrus gammamaculatus*

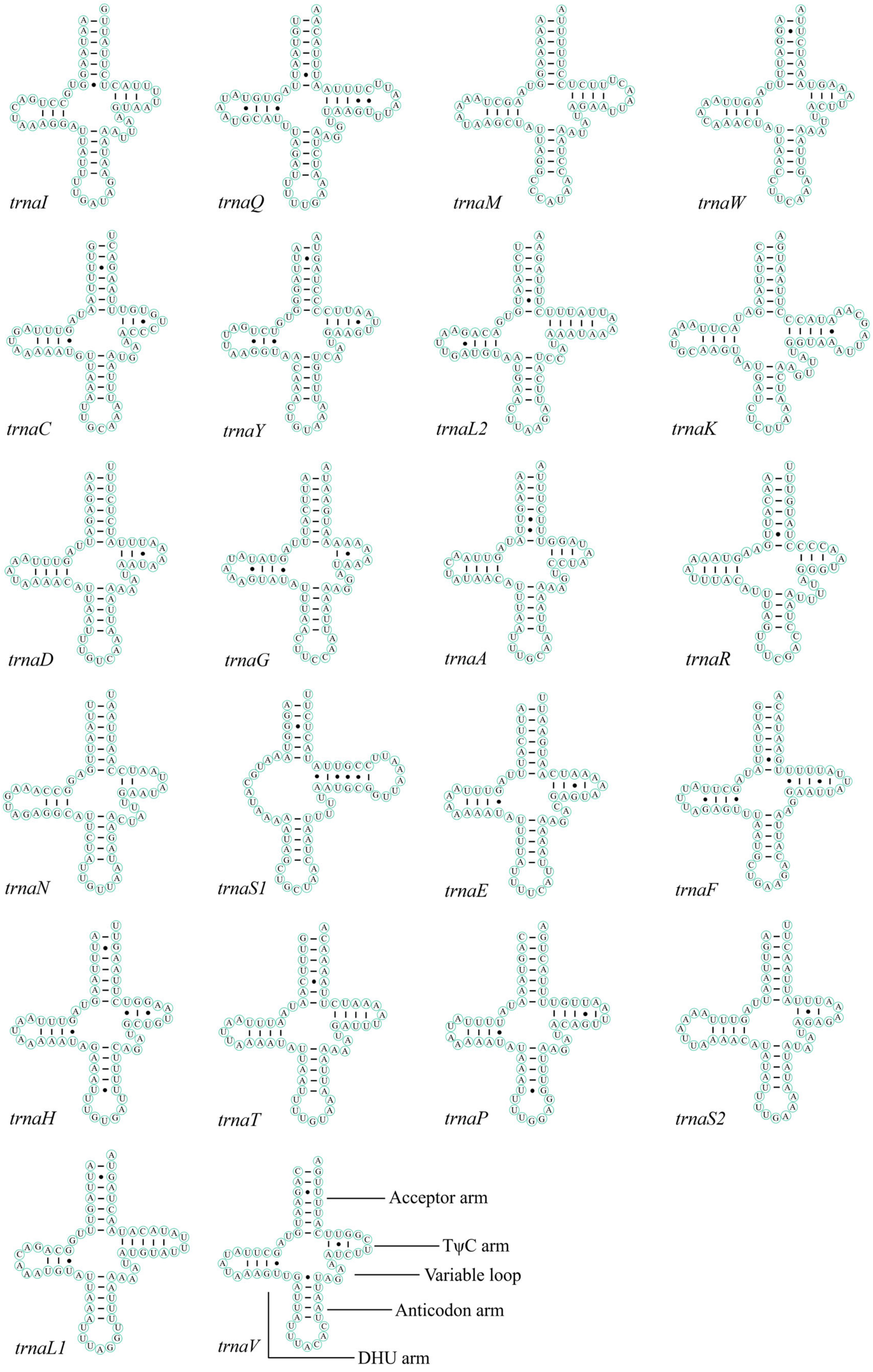

Figure S6. Predicted secondary cloverleaf structure for the tRNAs of *Antialcidas floripennae*

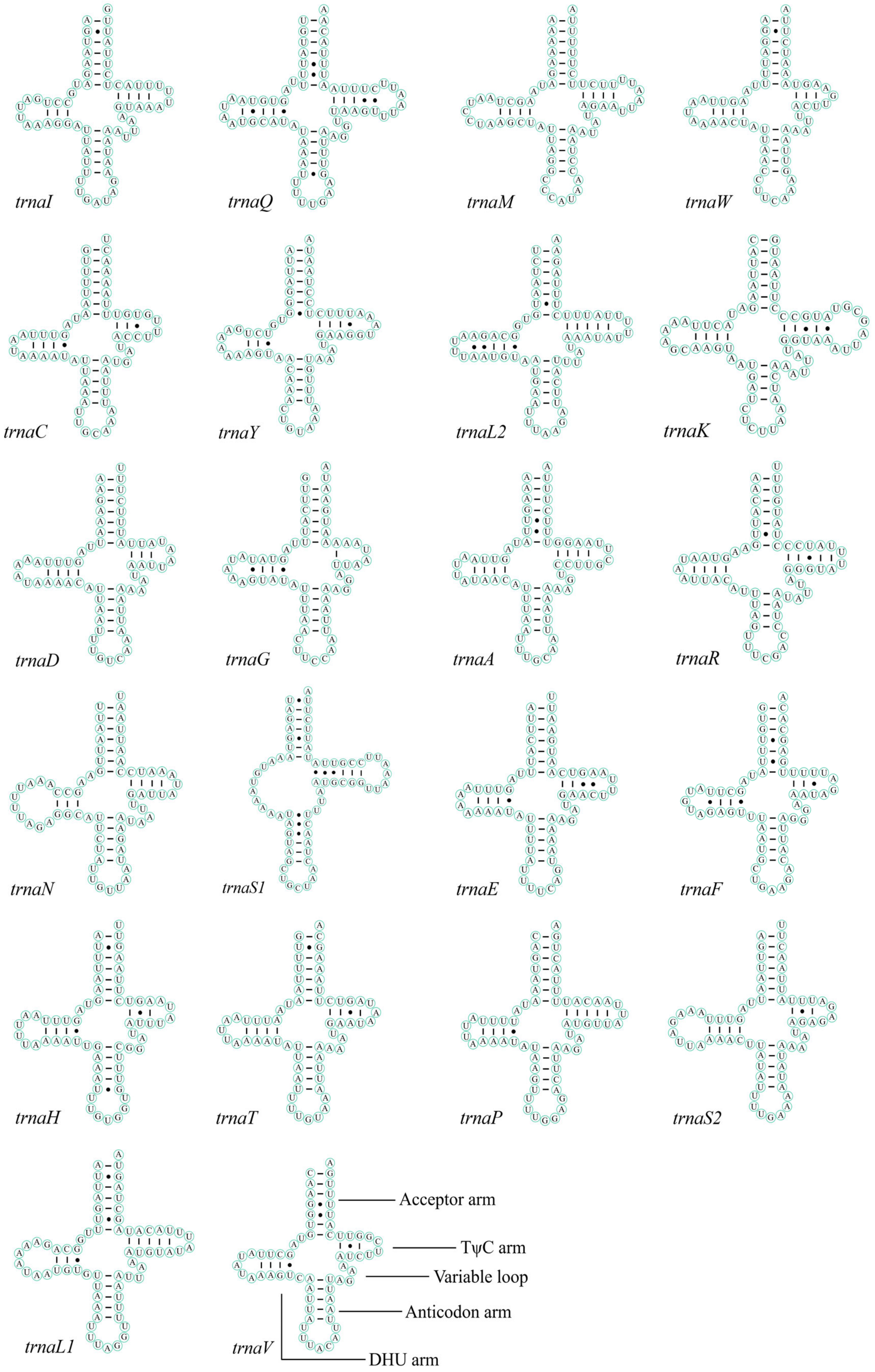

Figure S7. Predicted secondary cloverleaf structure for the tRNAs of *Machaerotypus stigmosus*

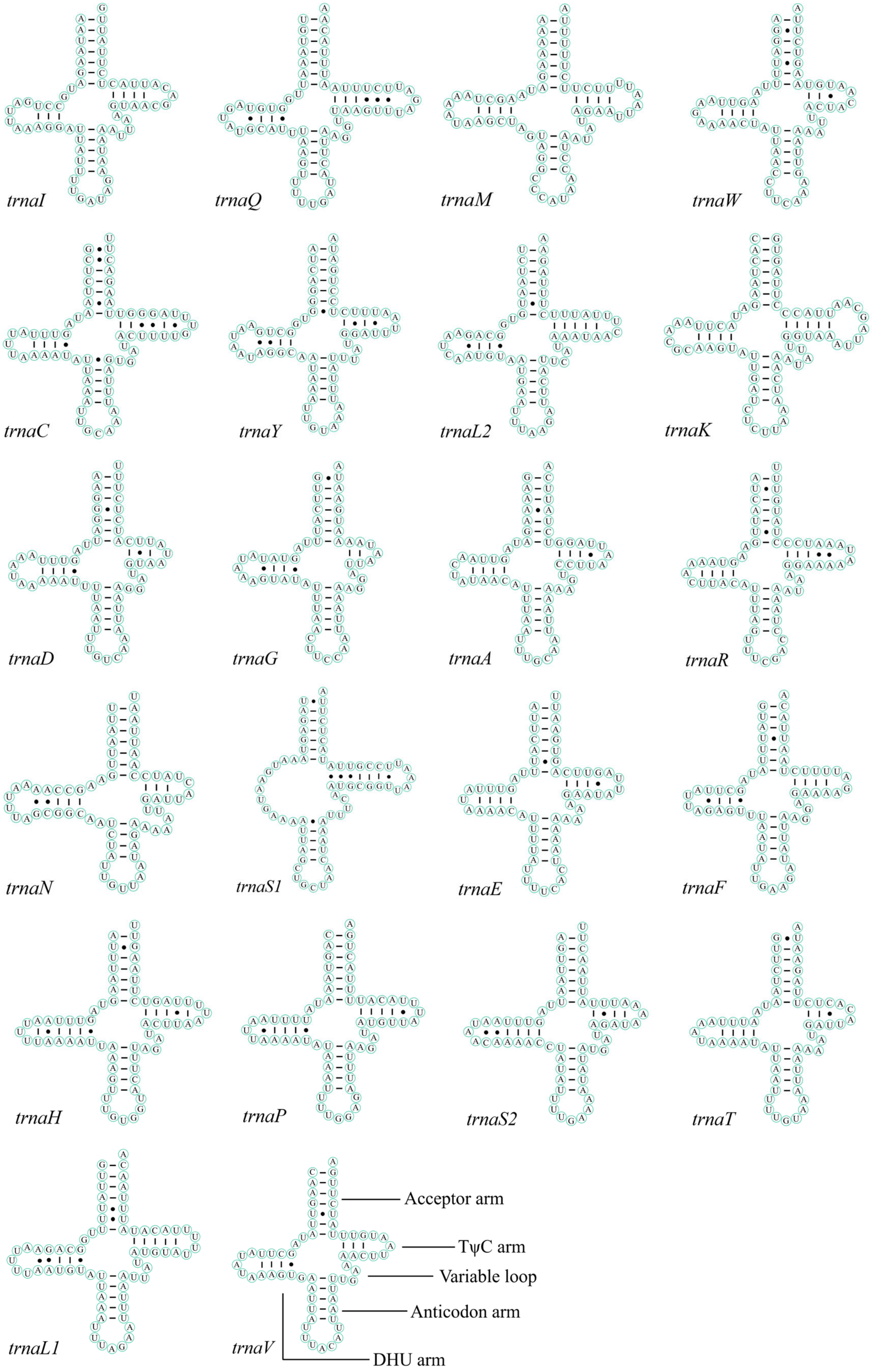

Figure S8. Predicted secondary cloverleaf structure for the tRNAs of *Centrotus cornutus*

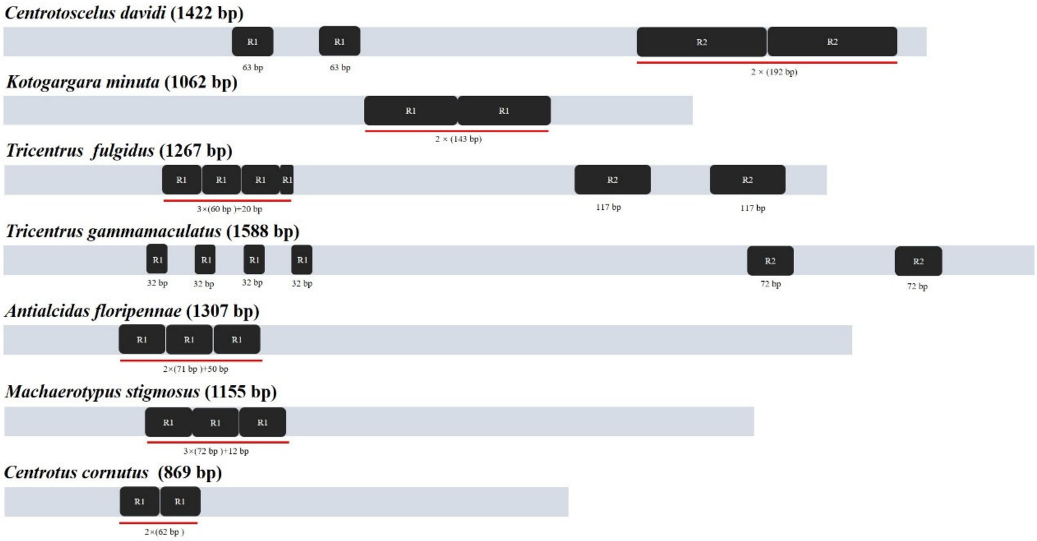

Figure S9. Features present in the control region of seven treehoppers

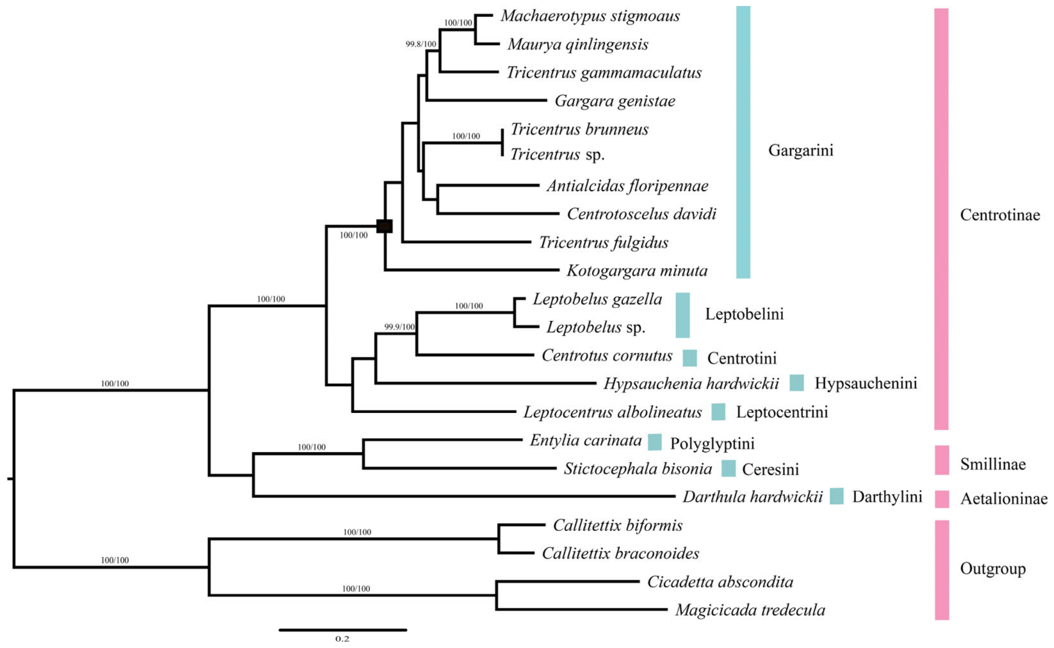

Figure S10. The phylogenetic tree of treehoppers was inferred using maximum likelihood (ML) analysis based on the amino acid sequences of the 13 protein-coding. Bootstrap percentage (BP) is indicated on branches. The black box indicates the branch of Gargarini tribe.

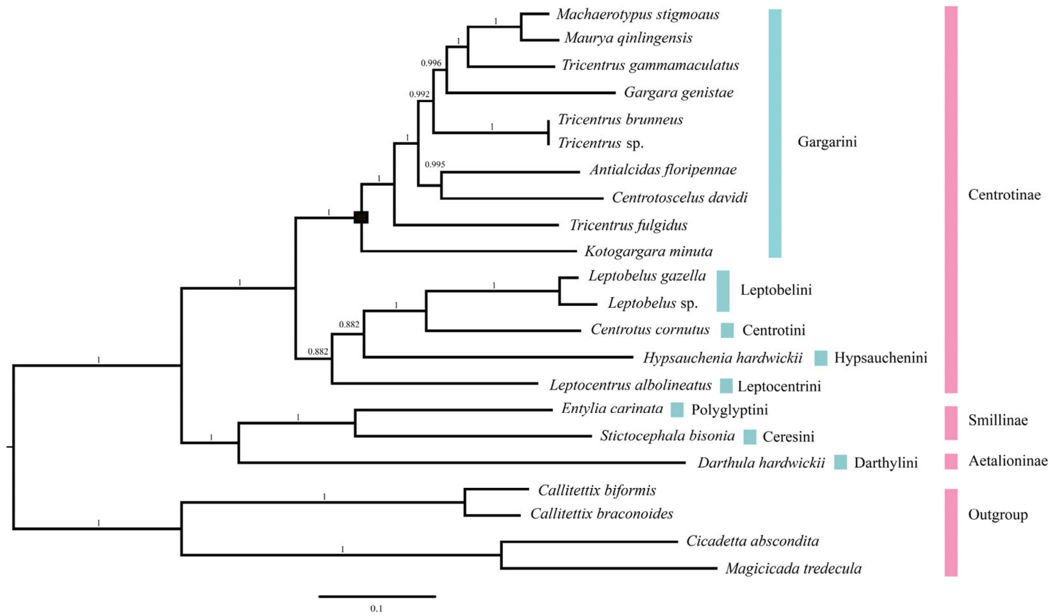

Figure S11. The phylogenetic tree of treehoppers was inferred using Bayesian inference (BI) analysis based on the amino acid sequences of the 13 protein-coding. Bayesian posterior probabilities (BPPs) are indicated on branches. The black box indicates the branch of Gargarini tribe.
